# Challenges of Integrated Home-Based Palliative Care Services for Cancer Patients during the COVID-19 Pandemic: A Qualitative Content Analysis

**DOI:** 10.1177/10848223221134780

**Published:** 2022-11-16

**Authors:** Zahra Alizadeh, Camelia Rohani, Maryam Rassouli, Mahnaz Ilkhani, Maryam Hazrati

**Affiliations:** 1 Shahid Beheshti University of Medical Sciences, Tehran, Iran; 2 Department of Health Care Sciences, Palliative Care Center, Marie Cederschiöld Högskola, Stockholm, Sweden; 3 Deparment of Community Health Nursing, School of Nursing and Midwifery, Shahid Beheshti University of Medical Sciences, Tehran, Iran; 4 Cancer Research Center, Shahid Beheshti University of Medical Sciences, Tehran, Iran; 5 Deparment of Medical-Surgical Nursing, School of Nursing and Midwifery, Shahid Beheshti University of Medical Sciences, Tehran, Iran; 6 Community Based Psychiatric Care Research Center, Shiraz University of Medical Sciences, Shiraz, Iran

**Keywords:** cancer, COVID-19, home care services, palliative care, hospital to home

## Abstract

Given the situation of cancer patients as vulnerable patients and the threat of COVID-19 in the society, integration of home-based palliative care services into the healthcare system is essential. The aim of this qualitative study was to explore the current barriers of integration of palliative care services from hospital to home for cancer patients during the COVID-19 Pandemic and to provide suggestions to resolve them. Semi-structured interviews were conducted with 25 stakeholders in the healthcare system, including health policy makers, healthcare providers, clinical home healthcare experts, home healthcare researchers, university faculty members, clergy, family caregivers, and cancer patients. Data were analyzed using directed content analysis method based on the World Health Organization Public Health Strategy for Palliative Care. Challenges were extracted in 4 main categories, containing education barriers (3 subcategories), implementation barriers (9 subcategories), policy barriers (5 subcategories), and drug availability barriers (2 subcategories). Based on the results, removing the barriers and establishing a strong infrastructure for home-based palliative care services is recommended in the healthcare system by concentrating on 4 essential factors, that is, utilizing a coordinating nurse during the process of patient’s hospital discharge, establishment of connecting outpatient palliative care clinics to home healthcare centers, access to palliative care tele-medicine and development of a comprehensive and flexible home-based palliative cancer care model in our context.

## Introduction

Cancer control is one of the priorities of World Health Organization (WHO) health programs. A comprehensive cancer control program includes screening, early detection, care provision for cancer survivors and palliative care.^1^ According to the WHO, palliative care is an approach that improves the Quality of Life of patients with life-threatening illness and their families by prevention and relief of symptoms through early detection, careful assessment and treatment of physical, psychosocial, and spiritual problems.^2^ A multidisciplinary team, including physicians, nurses and other healthcare professions should meet the complex needs of cancer patients in different settings levels.^3^ Thus, providing an integrated healthcare services within a strong infrastructure can be helpful and a priority.^4^

The WHO explains that “integrated care is often contraposed to fragmented and episodic care, and it is used synonymously to terms like coordinated care and seamless care, among others. However, there is no unifying definition or common conceptual understanding of integrated care.”^5^ It can be said that it is a pervasive term, consisting of wide and multi-component ideas and principles that require coordinated care based on the individual’s needs.^6^ Transition of care is one of the integral part of integrated healthcare services.^7^ It is defined as a patient’s movement through the healthcare system from hospital care into primary care or vice versa.^8^ In the process of transition of care, home healthcare services are essential for cancer patients to achieve optimal health goals.^9^ In addition, most patients who need palliative care prefer to stay at home. Therefore, palliative care should be provided as part of primary care in the community.^10^

But transition of palliative care services from hospital to the community is a challenging process.^11^ In this process, planning for continuity of cancer care during hospital discharge is essential. A review of the literature shows that there are several barriers for successful transition of care, including an inappropriate transition time, lack of proper health insurance, and insufficient knowledge about transition of care.^12^ In addition, medical and medication errors, inadequate communication, and deficiencies in care coordination have been reported during the transition of care.^13^

However the spread of the COVID-19 in the current situation, has caused a massive shift in the distribution of healthcare services in Iran, and it cannot be ignored. As, cancer patients are among the high-risk groups, excluding their unnecessary visits at hospitals has a great effect on reducing their risk of infection and creating peace of mind for the patients and their families.^14^ So far, there has been presented a very limited data on the impact of the COVID-19 on the process of integrated care.^15^ In Iran, with the start of the spread of the COVID-19 disease, patients suffering from chronic diseases, especially cancer, refused to go to hospitals and clinics due to the high risk of infection and preferred to receive healthcare services at home. Therefore, this study was conducted to explore the current challenges in integration of palliative care services from hospital to home for cancer patients during the COVID-19 Pandemic, and to provide suggestions to resolve the challenges.

## Theoretical Framework

**Figure 1.**
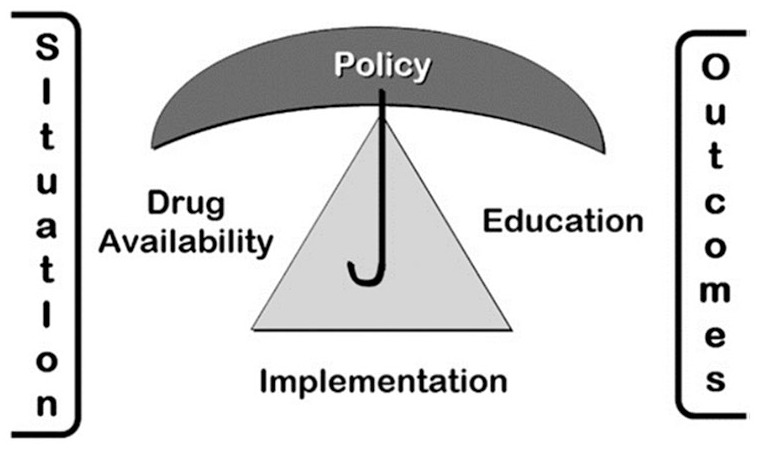
WHO public health model.^16^

The theoretical framework of this study is based on the WHO Public Health Strategy (PHS) for Palliative Care.^16^ This strategy originates from the Public Health Model (PHM) (Figure 1). In 1990, the WHO established the PHS to integrate palliative care into health care systems. This strategy contains recommendations and guidelines to governments on how to integrate national palliative care and cancer control programs into the health care system of a country and change the experience of patients and families. This strategy has 4 aspects: (1) appropriate policies, (2) adequate availability of drugs, (3) education of health care workers and the public, and (4) implementation of palliative care services in all levels of the community.^16^

## Healthcare Profile of the Country

A glance at the healthcare profile of Iran shows that there is a national public healthcare network and a private sector which are governed by the Ministry of Health and Medical Education (MOHME). The national public healthcare is called Primary Health Care (PHC) network and covers both urban and rural areas in the country.^17^ The private sector also plays a significant role in healthcare provision, where it mainly focuses on secondary and tertiary prevention in urban areas.^18^

Evidence shows that both palliative care and home healthcare centers are progressing in Iran. A “comprehensive national program” for providing palliative and supportive care for cancer patients has been started by the National Cancer Research Network in the MOHME from June 2011. Furthermore, a “palliative care committee” was established in the MOHME with the aim of integrating palliative care into the healthcare system in 2013.^19^ Moreover, the current situation shows that home healthcare centers are one of the important components of the healthcare system in Iran. There are 704 licensed home healthcare centers in 30 provinces of Iran, and they are progressing and developing. Right now, the private sector has authorities for home healthcare centers. Also, several university hospitals, assisting the private sector and deliver home healthcare services to chronic patients after hospital discharge.^20^

This study was planned to integrate home-based palliative care for cancer patients from hospital to the community. Based on the structure of our healthcare system, we planned to integrate the palliative cancer care from hospital to the network of the PHC, and private home healthcare system in the country.

## Research Question and Objective

The study research question was: “What are the experiences and views of the stakeholders (health policy makers, healthcare providers, clinical home healthcare experts, home healthcare researchers, university faculty members, clergy, family caregivers, and cancer patients) of the barriers of integrating home-based palliative cancer care services into the healthcare system during the COVID-19 pandemic?

The objective of the study was to explore the experiences and views of stakeholders about current barriers for integrating home-based palliative care services of cancer patients into the healthcare system during the COVID-19 pandemic based on the WHO strategy.

## Methods

### Study Design

This is a qualitative content analysis study for integration home-based palliative care services of cancer patients into the healthcare system. It is part of a Health Policy and Systems Research for developing a home-based palliative cancer care model in Iran. This study was carried out from March 1 to June 30, 2020 according to the needs of the society due to the spread of the Corona disease and the disruption of the palliative care for cancer patients after hospital discharge. In Iran, the first- case of the COVID19 was introduced on February 19, 2020 and after that on March 1, the total number of infected cases was 978, and by June 30, 227,662 cases were reported. Also, on June 1, the total number of deaths was 66, and on June 29, 10,670 mortality cases were reported in the country.^21^ The WHO declared the Corona pandemic on March 11, 2020.^22^

The ethical code of the study was obtained from the Research Ethics Committee of the university (IR.SBMU.PHARMACY.REC.1397.096).

### Participants

The participants were selected by purposive sampling up to the data saturation. They were 25 stakeholders, including health policy makers (3 persons), healthcare providers (3 persons), clinical home healthcare experts (7 persons), home healthcare researcher (one person), university faculty members (3 persons), clergy (one person), family caregivers (4 persons), and cancer patients (3 persons). They were selected from MOHME, 3 large medical universities, and 2 important centers with home healthcare services for cancer patients and palliative care, including 1 university hospital and a private cancer prevention and control center (MACSA) in Tehran, the capital of Iran. In this study, key informants were identified and selected from these organizations based on the inclusion criteria to obtain in-depth and rich information.

Cancer patients were eligible in this study if 1 year or more had passed from their diagnosis and they had received home-based palliative care services at least 6 months. Family caregivers included in the study, if they involved directly in the caring of their cancer patient at home at least 6 months. In addition, inclusion criteria for selection of the rest of participants were, having experience and expertise in the cancer and palliative care field and involved with home care for cancer patients at least 5 years.

### Data Collection

Data were collected using in-depth semi-structured face to face or telephone interviews with participants. All interviews were conducted by the first author in a quiet environment. The time of interviews for each participant was between 20 and 60 minutes. Before beginning the interview, the first author described the purpose of the study and obtained permission for the interview and recording. Participants were informed that their participation in the study was voluntary and can withdraw whenever they prefer. Semi-structured interviews were started with different type of questions for various types of participants. For example: “what is your experience of transitional care of cancer patients from hospital to home? “what is your experience of integrated home-based palliative care for cancer patients in the current situation of our healthcare system?,” “what are your experiences of the barriers or challenges of transition of palliative cancer care services from hospital to the home or vice versa in the healthcare system?,” “how is it about the barriers or challenges with the integration of home-based palliative care services into the PHC network and home care system in the current situation?.” Example questions for patients and family caregivers were: “what caring challenges do you experience during the transition from hospital to home?,” “what type of healthcare services do you need when your patient take a care at home?” and “what is your experience of receiving home palliative care services?.” During interviews, probing questions such as “can you explain more?” and “can you give me an example?” were asked to access to deep experiences of participants.

### Data Analysis

A directed content analysis method was used for data analysis by the approach of Hsieh and Shannon^23^ within the framework of the WHO PHS for Palliative Care.^16^ All recorded interviews were transcribed verbatim immediately and the whole of each interview was read several times to reach a comprehensive understanding of its content. Subsequently, meaning units and initial codes were extracted. Data analysis was done using MAXQDA-10.

### Rigor of the study

The criteria of Guba and Lincoln were used for rigor of the study, containing credibility, confirmability, dependability, and transferability.^24^ For data credibility, we used the method of prolonged engagement with participants and spending time to collect and analyze the data. Also, the research team members independently reviewed all the analyses. The analyses audited by 5 external referees (including 2 faculty members from 2 nursing schools with doctoral degrees in nursing and qualitative studies experts as well as 3 doctoral nursing students from 2 schools with skills in qualitative studies). In addition, dependability was achieved through systematic recording of the research process, and confirming examples of codes by 2 qualitative researchers. Moreover, the extracted codes were given to 3 participants to confirm the accuracy of the extracted codes. All stages of the study, were described in detail for confirmability. By registering different participants and describing the characteristics of them in terms of knowledge, experience, responsibility, length of working period, age and gender, transferability of the data was provided to access the experiences of the participants in a wide range.

## Results

Twenty-five stakeholders were interviewed, including 14 women and 11 men with the mean age of 41.24 ± 5.67 years. The mean duration of interviews was 31.33 ± 6.25 minutes. Table 1 shows the demographic information of the participants.

**Table 1.** Demographic Characteristics of Stakeholders (n = 25).

Variables	Number (%)
Sex	
Male	11 (44)
Female	14 (56)
Education	
Specialist physician and or PhD.	11 (44)
Master of Science (MS)	3 (12)
Bachelor of Science (BS)	7 (28)
College	4 (16)
Stakeholders	
Health policy maker	3 (12)
Healthcare provider	3 (12)
Home healthcare researcher	1 (4)
Clinical home health care experts	7 (28)
University faculty member	3 (12)
Clergy	1 (4)
Family caregiver	4 (16)
Patient	

A total of 546 codes were extracted from analyses of all interviews. Extracted codes were classified into the 4 main categories and 26 subcategories. Four main categories based on the PHS, included “education barriers,” “implementation barriers,” “policy barriers” and “drug availability barriers.” A sample of participants’ quotations for each subcategory was shown in Table 2.

**Table 2.** The Results of the Qualitative Content Analysis.

Categories	Subcategories	Quotations
Education barriers	Insufficient awareness of the public	“Public awareness about palliative care and home care is not enough, Radio and TV, as informative media, should be very active specially in Corona time and support such healthcare services. . . .the State of Welfare Organization should be involved in providing necessary information about healthcare services and preferences for cancer patients at home.” (Participant 1, Health Policymaker)
	Culture building	“In the public sight, there is no difference between care and treatment, and the hospital is the right place for both; a change in this mindset requires culture-building. Most people want their patient to be in the hospital until the last minute, and they are not willing to transfer him/her to home. Corona crisis and vulnerability of patients for being in the hospital, has made the needs of the society greater” (Participant 21, Head of the Home HealthCare Center)
	Insufficient trained healthcare providers	“The novelty of applying the concepts of palliative care and spiritual care at home in our society and the shortage of a strong educational curriculum in the university, are important reasons for insufficient trained healthcare providers.” (Participant 14, Nurse)
Implementation barriers	Access to a specific schedule of hospital discharge for cancer patients	“Some patients need to be referred to home care centers after hospital discharge, while some patients don’t need. Therefore, cancer patients should have a specific discharge schedule for hospital discharge and returning home, including follow-up and telephone counseling during 24 hours of the day, a list of common patients’ problems, warning signs, medications, and emphasis on self-care training and visiting time (Participant 5, Researcher in the Home Care)
	Establishment of a strong infrastructure	“At first we need policy making in this area, actually we need a specific infrastructure for home-based palliative care in our society which should be flexible in the time of crisis like COVID-19 pandemic. . . . There are several barriers to set up home care centers with specialized palliative care services. . ..not enough facilities, insufficient budget allocation, insufficient training and shortages of trained staff, we need connected palliative care clinics to home healthcare centers and hospice, challenges for access to medicines and insurance coverage at home, also access to a specific electronic platform, are important current challenges for home-based palliative care in our healthcare system.” (Participant 1, Health Policymaker)
	Involving graduated community health nurses	“We need specialized personnel in the field of home-based palliative care. Community health nurses with specialized training in the field of home healthcare, can be active in this specific field at home. Unfortunately they are not involved in the care at home and community in our society. (Participant 7, Factually Member)
	Access to packages of home-based palliative cancer care services	“In order to increase quality of life of cancer patients, they need to receive comprehensive and standard healthcare services after hospital discharge. A specific care track for cancer patients and their family prevents their confusing after hospital discharge; this will be possible by access to comprehensive packages of healthcare services with specific guidelines for cancer patients and palliative care at home and end of life care. . . . . . now we need special packages for palliative care in cancer patients during the disease of Corona also.” (Participant 13, Home HealthCare Nurse)
	Cohesion and professional solidarity in the home-based palliative care team	“in my opinion when we talk about palliative healthcare team, cooperation and coordination between the team members are essential. We need to have a national symposium or meeting with our home care colleagues.” (Participant 17, Home HealthCare Nurse)
	Necessity of cancer patient’s follow-up after hospital discharge	“After the mastectomy, I was discharged from the hospital with a note containing only a few lines about the complications after surgery, the medications and the time of the next visit to the doctor . . . . After that, no one called me to follow up my condition while I need to it.” (Participant 22, Patient)
	Poor transitional care from hospital to home	“Poor transfer of care from the hospital to home care centers, the lack of patient follow-up after hospital discharge and 24-hour telephone consultation, and the lack of an electronic platform for recording of patient’s data and transferring information to home care centers can be an obstacle to safe transfer from hospital to home. ” (Participant 1, Health Policymaker)
	Access to a coordinate nurse	“The existence of a coordinator to coordinate healthcare services and plan for integrated healthcare services is an important issue for reducing patient readmission at hospital.” (Participant 9, Home HealthCare Nurse)
	Insufficient involvement of patients and their family in caring	“One of the principles of transitional care from hospital to home, is the involvement and participation of patients and their families in the process of caring and recieving education and supportive care.” (Participant 7, Faculty Member)
Policy barriers	Weaknesses in legal-security issues for home care	“Providing security in home visits, is another essential challenge during delivering palliative home care. Since, the visit takes place at home in an informal place for caring, it can pose risks to the family and healthcare providers, so it makes sense if healthcare providers would be very cautious or are not willing to enter that area . . . ”(Participant 6, Home HealthCare Nurse)
	The loss of health insurance coverage for home healthcare services	“A patient with cancer should not be in charge of self-protection. When a patient come back home from hospital and go under caring of home healthcare centers, he/she cannot be under coverage of the health insurance. There is no health insurance for coverage of home healthcare services in our healthcare system.” (Participant 2, Health Policymaker)
	The lack of liability insurance for home healthcare providers	“Working alone and without liability insurance in home care centers, produce a lot of stress for the home care nurses and makes working conditions difficult for them.” (Participant 17, Home HealthCare Nurse)
	Non-transparent payment medical tariffs for home health care services	“The lack of a transparent tariffs for healthcare services provided at home, should be taken into account. In some cases, the lack of tariffs for these services has led to families’ paying higher costs and taking action subjectively.” (Participant 14, Nurse)
	The need for a flexible home-based palliative care model	“The existence of a flexible home palliative care model, assist to plan for transition of healthcare of the patient from hospital to home in accordance with the healthcare system, especially in the current situation when we have the Corona disease and there should be specific precautions at hospital, home and community for disease prevention.” (Participant 8, Expert of Home Care in the Ministry of Health)
Drug availability barriers	Limitations for oral and non-oral narcotic medications at home	“The biggest challenge regarding medication is the lack of oral morphine in our country. Another challenge is related to the prescription of the narcotics at home, for example in our home care center, specialist physician can prescribe narcotic medicines and a general practitioner cannot. . . . in some cases, our patients even have problems with providing injectable morphine also. . . it is not easy to find morphine, especially with spreading of Corona and limited access to the medicines and shortages. . . . our patients problem is more than earlier. It is advised to have clear regulations and rules for prescription of narcotics. Also, there must be better access to narcotics for cancer patients at home. ” (Participant 3, Physician)
	No legal permission for nurse prescribing	“In our healthcare system, nurse prescribing is not legal. If a patient get diarrhea, a home healthcare nurse knows that the patient needs Dicyclomine or Loperamide in this condition. . . but our nurses have no license or permission for prescription of medicines, therefore they cannot prescribe at home and manage symptoms of cancer patients. ”(Participant 5, Researcher in the Home Care)

### Category 1: Education Barriers

Education is an important part of providing high quality healthcare services. In this area, 3 sub-categories extracted: “Insufficient awareness of the public,” “Culture building” and “Insufficient trained healthcare providers” (Table 2).

### Category 2: Implementation Barriers

This category includes 9 subcategories: “Access to a specific schedule of hospital discharge for cancer patients,” “Establishment of a strong infrastructure,” “Involving graduated community health nurses,” “Access to packages of home-based palliative cancer care services,” “Cohesion and professional solidarity in the home-based palliative care team,” “Necessity of cancer patient’s follow-up after hospital discharge,” “Poor transitional care from hospital to home,” “Access to a coordinate nurse,” and “Insufficient involvement of patients and their family in caring” (Table 2).

### Category 3: Policy Barriers

The purpose of policy-making is to determine general policies in various health programs for patients and their families as well as their healthcare providers. Five sub-categories of policy-related barriers includes: “Weaknesses in legal-security issues for home care,” “The loss of health insurance coverage for home healthcare services,” “The lack of liability insurance for home healthcare providers,” “Non-transparent payment medical tariffs for home healthcare services,” and “The need for a flexible home-based palliative care model ” (Table 2).

### Category 4: Drug Availability Barriers

According to the participants’ statements, appropriate access to medication was one of the most important components that affects the quality of palliative care services at home. This category includes the 2 sub-categories of “Limitations for oral and non-oral narcotic medications at home” and “No legal permission for nurse prescribing” (Table 2).

## Discussion

The present study aims to explore the experiences of stakeholders about current challenges in integration of home-based palliative care services of cancer patients in the healthcare system of Iran during the spread of the COVID-19, and to provide suggestions to resolve the challenges. The challenges were extracted in 4 categories based on the WHO strategy for palliative care, including “education barriers,” “implementation barriers,” “policy barriers,” and “drug availability barriers.”^16^ Given the situation of the spread of the COVID-19 disease in the world, cancer patients as one of the vulnerable groups, are threatened by the disease. Thus, caring in a safe setting such as home with the aim of reducing infection and keeping patients safe from the potential dangers of COVID-19 and other infections at the hospital was important.^25,26^

The first extracted category of challenges for integration of home-based palliative cancer care services in the healthcare system, was education barriers. A glance at the details of the results shows that one of the challenges, is insufficient knowledge of the public and cultural barriers in the society. Low level of knowledge in the public and their misconceptions about home-based palliative care can bring a negative impact on the acceptance of these services in the society.^27^ Therefore, increasing families awareness and changing their attitudes is recommended. In this regard, media, including radio and television can be very helpful by holding scientific discussions to increase public awareness. Also, the activities of responsible organizations such as the MOHME and the Welfare Organization can have a great impact on movement toward positive attitudes and increasing awareness of the families in the society.

The second and third categories, implementation and policy barriers, involved serious challenges. Implementation of palliative care at home requires to establish a strong infrastructure for home-based palliative care. This infrastructure should be adapted with the healthcare system and current crisis. Infrastructure includes the appropriate environment and supporting factors such as equipment, communication technologies, access to analgesic medications, proper educational curriculum for healthcare personnel and access to financial resources.^28,29^ In addition to trained qualified staff and allocating resources, health systems should prepare appropriate packages for home-based palliative care services with specific guidelines in accordance with the context.^30^ In this regard, the MOHME has developed a series of educational packages for home care for 6 chronic diseases, including “cancer and palliative care.” But in the current situation, there is a need for the development or renewal of educational packages with focus on prevention precautions for COVID-19 in cancer patients. Moreover, the MOHME has started a periodical national palliative care education program several years ago.^20^ Nurses and physicians are invited to this program and this program should be continued and developed in our context.

Now, when the world has been confronted with the COVID crisis, this situation is more risky for cancer patients due to the high-risk of infection in the hospital and travel bans. Establishing an electronic platform and applying “telemedicine” to provide palliative care at home, is a critical part of home-based palliative care infrastructure.^31^ Peckham et al reported that having 24 hours/7 days support, through an advanced technology, facilitates the access to healthcare services and creates a sense of security for the patient and family, which leads to greater coordination of care, prevention of complications and readmission of the patients to the hospital.^32^ Therefore, in the current situation, establishing of “palliative care tele-medicine” in the healthcare system is essential. It is necessary to consider the appropriate location and infrastructure to set up an electronic platform. This platform is used for registering the medical records of cancer patients during hospitalization, discharge, triage, and telephone counseling.^33^ The results of a previous study showed that after hospital discharge, healthcare providers lose their communication with cancer patients, and the needs of patients and their families remain unmet.^34^ During hospital discharge, nurses should evaluate patients’ self-care, their mobility, transition of their care and the development of an integrated care program.^35^ Integration of palliative care early in the cancer treatment has been recommended in the 2016 clinical guideline of the American Society of Clinical Oncology Clinical Practice Guideline (ASCO).^36^ The medical literature shows positive results from outpatient palliative care clinics in different parts of the world.^37^ The outpatient clinic deliver a specific level of palliative care services such as a brief consultation, a concurrent care with alliance of the patient’s primary physician^35^ as well as referral process of the patients.^36,38^ These clinics are also responsible for post-discharge follow-up, continuity of care from hospital, supervision of patients’ medication and response to patients and their families questions and patients’ concerns after hospital discharge and returning home and the referral process of patients to home care centers.^37,39,40^ Based on the current situation of the healthcare system in Iran, developing of outpatient palliative care clinics at hospitals and their connection to the network of the PHC and home care centers, is recommended. Moreover, it is obvious that coordination and communication of healthcare personnel at hospitals and in the home care centers can improve the quality of palliative care services.^33^

In time of the COVID-19 Pandemic, all accessible resources should be used. Graduated nursing students in the field of community health nursing, are one of the existing resources who can play an important role in providing home healthcare services. According to the current rules, they are allowed to be active only in hospitals, thus the roles of the community health nurses should be expanded in our healthcare system by health policy makers.^41^ Their roles should be expanded in the community to deliver home care services in the network of PHC. Evidence shows that the development of specialized roles of community health nurses in primary healthcare settings has taken place in developed countries many years ago.^42^ Therefore, it is suggested that in order to cover the palliative care needs of cancer patients at home to pay attention to reform and role expansion of community health nurses in the society.

Also, our results showed that attention to the legal-security issues for delivering safe home care services, and coverage of health insurance for these services, are important. Furthermore, access to appropriate models and guidelines to provide a comprehensive and flexible home-based palliative care services is necessary. We need an adapted model during the COVID-19 Pandemic in our society. This model can assist the integration of home-based palliative care services into all levels of the PHC in our healthcare system. One part of this model for hospital discharge and transition of cancer care to home in urban areas has been developed and published elsewhere.^33^ In a comprehensive care program, the continuity of care, providing necessary information and communication between healthcare providers and the patient and his/her family should be clear. Also, it should be possible to contact the healthcare personnel 24 hours a day.^33^

The last extracted category of challenges was drug availability. Another issue is related to prescription and application of the medicines at home. Pain control in cancer patients is important, especially during the pandemic crisis due to limited transportation and infection in the hospital.^43^ In Iran for pain control, there is only access to injectable analgesics not oral. Also, opioids use has a cultural stigma, and sometimes insufficient knowledge of healthcare team members, making the situation worse.^44^ Prescription at home is very important. Authorized persons should be used for prescribing and the type and list of the medicines should be very clear. As, nurses have no permission for prescribing of medicines at home, the most important step is to legalize nurse prescribing at home for nurses in Iran. According to the results of the previous studies, there is nurse prescribing in developed and some developing countries.^28^

In summary, although home-based palliative care is an essential element of the continuation of cancer care, there are several barriers in the areas of education, implementation, policy and drug availability, particularly in the crisis of the COVID-19 Pandemic. These barriers prevent the integration of home-based palliative care services into the healthcare system in our context. Through evolving the current palliative care infrastructure, it is recommended to develop connecting outpatient palliative care clinics to home healthcare centers and to provide access to palliative care tele-medicine. A comprehensive and flexible home-based palliative care model in our context can decrease readmission and duration of hospital stay for cancer patients. Our recommendations during the COVID-19 pandemic can be a good example for international readers with similar or even different healthcare context.

### Limitations and Strengths of the study

The strengths of this study are using WHO strategy to extract the barriers of home-based palliative care, and exploring a clear roadmap for removing barriers and transition of palliative cancer care from hospital to home and integrating of the services to the healthcare system of Iran. One of the limitations of this study was the limited access to specialized and experienced participants in the field of home care. Also, inadequate infrastructure of the home healthcare system in our society and the coverage of this system by the private sector, have created some limitations in our study. Although, the interviews were conducted in a way that elicited the most information from our participants and avoided potential interview bias, there was still a possibility of interview bias and/or selection of biased experts.

## Conclusions

In this study, the barriers of the integration of home-based palliative cancer care services to the current healthcare system were explored during the COVID-19 pandemic and according to the WHO strategy. Four barrier categories were extracted: education, implementation, policy and drug availability barriers. For removing the barriers, it is important to set up a strong infrastructure for home-based palliative care by concentrating on transitional care and connecting outpatient palliative care clinics to the home healthcare centers, and PHC network in our country. Utilizing a coordinating nurse during the process of patient’s hospital discharge, access to palliative care tele-medicine and a comprehensive and flexible home-based palliative cancer care model can be effective. Our recommendations during the spread of the COVID-19 disease can be a guide for international readers with similar or even different healthcare system context. In the next study, the authors use these results and the rest of the phases of a Health Policy Systems Research to develop a comprehensive model of home-based palliative cancer care that is compatible with the healthcare system of Iran.
